# Treatment of toxoplasmosis: Current options and future perspectives

**DOI:** 10.1016/j.fawpar.2019.e00036

**Published:** 2019-04-01

**Authors:** Neda Konstantinovic, Hélène Guegan, Tijana Stäjner, Sorya Belaz, Florence Robert-Gangneux

**Affiliations:** a National Reference Laboratory for Toxoplasmosis, Institute for Medical Research, University of Belgrade, 11129 Belgrade, Serbia; b Univ Rennes, CHU Rennes, Inserm, EHESP, Irset - UMR_S 1085, F-35000 Rennes, France

## Abstract

Toxoplasmosis is a worldwide parasitic disease infecting about one third of humans, with possible severe outcomes in neonates and immunocompromised patients. Despite continuous and successful efforts to improve diagnosis, therapeutic schemes have barely evolved since many years. This article aims at reviewing the main clinical trials and current treatment practices, and at addressing future perspectives in the light of ongoing researches.

## Highlights

• Toxoplasmosis is a widespread zoonosis, which may have an important impact on human and animal health worldwide
• Treatment schemes remain unchanged since 20 years, as no new drug has been put recently on the market
• Among the few drugs currently available for treatment of toxoplasmosis, none of them is efficient on cysts
• Active research to discover new therapeutic targets is ongoing, and promising pathways have been identified, thus paving the way for future drug combinations to eradicate the parasite
• Immunomodulatory approaches could add value to prevent relapses in immunocompromised patients, but are still underdeveloped

## 1 Introduction

*Toxoplasma gondii* is one of the most successful parasites in the world, as about one third of the worldwide population is seropositive for toxoplasmosis (Montoya and Liesenfeld, 2004). The deleterious impact of primary infection during pregnancy, as well as the reactivation of the disease in immunocompromised patients are well known since decades, but, while many gaps have been filled in the diagnosis field, in epidemiology, or in the comprehension of parasite-cell interplay, few advances have been made in the treatment of toxoplasmosis. Congenital toxoplasmosis (CT) remains a considerable burden on global health, with an estimated equivalent of 1.2 million disability-adjusted life-years (DALY) for >190.000 annual cases (Torgerson and Mastroiacovo, 2013), with long-term neurological sequelae and/or visual impairment possibly expected in infected untreated children.

Whereas HIV-associated *Toxoplasma* reactivation has readily decreased in high income countries, after the introduction of highly active antiretroviral therapy, it is still very prevalent in low income countries. Wang et al. have recently calculated that >13 million HIV-infected people were *Toxoplasma*-seropositive worldwide, thus at risk for cerebral toxoplasmosis, with 87% of them living in sub-Saharan Africa (Wang et al., 2017).

Available options for toxoplasmosis chemotherapy are limited. The folate pathway, involved in DNA synthesis with the dihydrofolate reductase (DHFR) and dihydropteroate synthetase (DHPS) enzymes, is the main target of anti-*Toxoplasma* drugs. Pyrimethamine (PYR) and trimethoprime (TMP), two major drugs in the treatment of acute toxoplasmosis, both act on parasite DHFR, but are unable to distinguish it from the enzyme of the human host. Taken alone, they are not enough powerful, thus they must be associated in combination regimens with sulfonamides which block DHPS. Therefore, current treatment regimens have side effects due to myelotoxicity (not to mention more severe ones that can be life-threatening), and require discontinuation of therapy, or, more frequently, induce lack of compliance. This is a serious drawback, as patients (congenitally infected neonates, immunocompromised patients) usually need prolonged courses of treatment. Most of all, no current drug is able to eliminate *T. gondii* cysts from the infected host, which remain quiescent, provided that the immune system is strong enough to hamper their reactivation into tachyzoites.

Drug resistance in *T. gondii* is considered to be a negligible issue, compared to poor compliance and the spectrum of adverse events. However, failure of the long-term PYR-based treatment for congenital toxoplasmosis (CT), possibly due to the development of drug-resistant *T. gondii* strain, has been reported (Villena et al., 1998). Moreover, Silva et al. have recently isolated a sulfadiazine-resistant *T. gondii* strain from congenitally infected newborns in Brazil (Silva et al., 2017). Although limited by the slow multiplication of the parasite and the transmission routes of the disease, both preventing wide spread of a resistant strain, this finding shows that drug resistance development in *T. gondii* is possible.

Therefore, it is of great importance to identify novel potent candidates that would be well-tolerated in both pregnant women and newborns and would act on both tachyzoites and cysts. From a pharmacokinetics point of view, an ideal treatment should be bioavailable, should concentrate in the placenta but also distribute into the fetal compartment, it should cross the blood-brain barrier and diffuse into the central nervous system (CNS) and the eye compartment as well, to reduce number of cysts. Development of non-toxic and well tolerated drugs that would prevent reactivation, shorten treatment duration or even eradicate chronic toxoplasmosis would revolutionize current *T. gondii* treatment. Finally, such new drug should be affordable, so that it could be used in low income countries.

In this review, we will address the current treatments available for the treatment of congenital toxoplasmosis and for reactivation episodes in immunocompromised patients, and explore the various strategies to develop new anti-*Toxoplasma* drugs.

## 2 Current treatment options: which, how and for what benefit?

### 2.1 Congenital toxoplasmosis

There are two time points for the introduction of specific anti-*T. gondii* treatment: 1) prenatal treatment, aimed at prevention of materno-fetal transmission of parasites (MFTP) and/or reducing fetal damage, and 2) postnatal treatment, with the purpose of alleviation of clinical manifestations and/or prevention of long-term sequelae in the infected neonate.

However, the benefits of prenatal treatment has been diversely appreciated in the literature due to confounding factors (Robert-Gangneux, 2014), since it may depend, among others, on the type of treatment, the time of introduction after maternal infection, the dose regimens and duration. Therefore, a prerequisite is to know the accurate time of maternal infection, which is achievable only in countries with serological screening programs of pregnant women, i.e. a limited number of European countries. The benefit/risk ratio of postnatal treatment has also been questioned, especially in asymptomatic or subclinical infected patients, for whom the duration of treatment and the long-term benefits are still under debate (Petersen, 2007).

#### 2.1.1 Prenatal treatment

Women infected during pregnancy (or around conception) are generally offered spiramycin (SPI), a potent macrolide antibiotic that concentrates in the placenta, making it an ideal preliminary treatment option for the prevention of MFTP (Table 1). Due to the low rate of adverse effects, SPI is a comfortable treatment option while awaiting amniocentesis. Unfortunately, SPI is ineffective for the treatment of an established fetal infection, since it barely crosses the placental barrier (Robert-Gangneux et al., 2011).

**Table 1:** Recommended treatment options for congenital toxoplasmosis.

Clinical entity	Treatment	Regimen	Administration	Duration
Acute toxoplasmosis in pregnancy (no proof of fetal infection)	SPI^a^	1 g [3 million units] ×3/d	p.o.	Until amniocentesis (AF PCR result) and/or delivery^b^
Fetal toxoplasmosis	Combination 1 + 2 + 3		p.o.	Until delivery
	1. PYR	50 mg/d		
	2. SDZ	4–6 g/d		
	3. FA	50 mg/w (once/w)		
Neonatal toxoplasmosis	Combination 1 + 2+3^c^		p.o.	12mo
	1. PYR	2 mg/kg/d for 2d, then 1 mg/kg/d for 6 mo (or only 2 mo if asymptomatic) and then 1 mg/kg 3×/w for the last 6 mo (or 10 mo)		
	2. SDZ	100 mg/kg/d		
	3. FA	15 mg/w (3 × 5 mg)^d^		

SPI: Spiramycin; PYR: Pyrimethamine; SDZ: Sulphadiazine; FA: Folinic acid; p.o.: per os; h: hour; d: day; w: week; gw: gestational week; mo: month; AF: amniotic fluid.

a For infections diagnosed later than 14 gw, US and some European centres (e.g. Austrian) recommend immediate PYR + SDZ + FA treatment (that may be switched to SPI if AF PCR result comes out negative) (Maldonado, Read and Committee on infectious diseases, 2017; Dunay et al., 2018).

b If fetal infection was not diagnosed or even tested (late pregnancy infections).

c Elevated CSF protein concentration (> 1 g/dL) or retinitis indicate introduction of prednisolone 1 mg/kg (p.o.)

d Neutropenia indicates higher dosage (up to 20 mg/d), followed by 10 mg/d from one month of age (4.5 kg body weight) for 11 months.

PCR analysis of the AF samples from the 16th gestational week (gw) onwards allows for a treatment switch to PYR-based combinations, primarily PYR – sulfadiazine combination (PYR-SDZ) when a positive PCR result is obtained (Table 1). However, the PYR-SDZ combination is teratogenic and hence should be avoided during the first 14 gw, although this cut-off varies between countries (Dunay et al., 2018). Anyway, prenatal diagnosis is never performed before 14 gw, thus SPI treatment is the rule during the first trimester of gestation.

The protective effect of SPI has long been known. The classical study by Desmonts and Couvreur (Desmonts and Couvreur, 1974) reported an over 50% decrease in MFTP (45% in untreated vs. 22% in the treated group), but the results were biased by not taking into account the gestational age at seroconversion. Studies that followed were also in favor of prenatal treatment (Daffos et al., 1988; Patel et al., 1996). However, several observational studies published since 1999 cast doubt on the capacity of prenatal treatment to reduce the severity of CT, while acknowledging its role in reducing maternofetal transmission (Foulon et al., 1999; Gilbert, Gras and European Multicentre Study on Congenital Toxoplasmosis, 2003; Cortina-Borja et al., 2010). Further studies dealt primarily with the timing of the introduction of prenatal treatment vs. maternal seroconversion, with the drawback of merging data from countries with variable screening practices, thus leading to uncertainty on the time of maternal infection (Robert-Gangneux, 2014). The 2005 EMSCOT study reported 72% lower odds of intracranial lesions in infants born to mothers treated within 4 weeks after seroconversion (OR 0.28; CI: 0.08–0.75) (Gras et al., 2005). The SYROCOT 2007 meta-analysis of 1438 treated mothers revealed that initiation of prenatal treatment within three weeks after seroconversion led to a 52% reduction of MFTP compared with treatment introduced after 8 or more weeks, but reported no clear effect of prenatal treatment on the rate of clinical manifestations among infected newborns (Thiébaut et al., 2007). However, the effect of treatment on clinical sequelae was evaluated only on live born infants, and the study suffered from bias due to heterogeneity of data. On the other hand, Hotop et al. found delayed treatment (> 8w after seroconversion) to be a risk factor for symptomatic CT (Hotop et al., 2012). Of note, the introduction of monthly systematic screening for toxoplasmosis in pregnancy in France has resulted in an overall decrease in the rate of MFTP from 29% before 1992 to 24% after 1992, and an impact of immediate introduction of PYR-SDZ treatment on the reduction of clinical CT cases from 11% before 1995 to 4% after 1995 (Wallon et al., 2013). Austria, another European country with a decades-long national screening program for toxoplasmosis in pregnancy accomplished a fascinating reduction in MFTP among prenatally diagnosed and treated women – 9% in comparison to previously 51% in untreated women, according to the Austrian Toxoplasmosis Register data (1992–2008 period) (Prusa et al., 2015).

A recently published randomized, open-label phase-III clinical trial on the efficacy and compliance of SPI vs. PYR-SDZ in reducing the MFTP included 36 French centers with enrollment of 143 women who seroconverted in pregnancy during the 2010–2014 period. Proven fetal infection after amniocentesis >18 gw triggered a switch from treatment with SPI to PYR-SDZ up to delivery. The MFTP was lower in the PYR-SDZ group – 18.5% (12/65) vs. 30% in the SPI group (18/60). The efficacy of PYR-SDZ vs SPI was higher when the treatment was introduced within 3 weeks after seroconversion (OR 1.20 vs OR 0.03), suggesting a window of efficacy for the PYR-SDZ regimen to prevent MFTP after maternal infection. Besides, follow-up revealed abnormal ultrasonographic findings in 8.6% (6/70) fetuses in the SPI group (including 2 cases of severe CT leading to pregnancy termination) whereas none were observed in the PYR-SDZ group (*P* = 0.01) (Mandelbrot et al., 2018).

#### 2.1.2 Postnatal treatment

Postnatal treatment is started when the diagnosis of congenital infection is confirmed and aims at preventing or reducing clinical manifestations at birth and alleviating possible long-term sequelae or clinical relapses, mainly eye sequelae. A classical study that changed the general approach to post-natal treatment of CT was the 1994 Chicago Collaborative Treatment Trial (CCTT), which revealed an encouraging outcome of a year-long PYR-SDZ treatment in 120 infected neonates followed up between 1981 and 2004, significantly better than in untreated (or sub-optimally treated) historical controls. Even among children with severe presentations at birth, 80% had normal motor function, 64% did not develop new eye lesions, and none developed sensorineural hearing loss (McLeod McLeod et al., 2006). Actually, the CCTT also standardized the PYR-SDZ treatment regimen, and recommended that it should be administered continuously throughout the entire first year of a congenitally infected child; actualized recommendations have published recently (Maldonado, Read and Committee on infectious diseases, 2017) (Table 1).

The prognosis for infected children is improved by the introduction of PYR-SDZ treatment immediately after birth but is feasible only in centers offering prenatal diagnosis or neonatal screening (serology, CNS imaging and ophthalmological examination) (Peyron et al., 2017). Neonatal screening for CT is systematically conducted in Massachusetts and New Hampshire (USA) and in Brazil, but is usually performed only on demand in other countries. Early treatment is equally important in asymptomatic and subclinical newborns, as it reduces the onset of clinical manifestations (Wilson et al., 1980; Phan et al., 2008), and in symptomatic children where it is expected to ease the symptoms and to reduce the long-term sequelae (cerebral calcifications, retinal disease and even microcephaly and hydrocephalus) (McLeod et al., 2006). Treatment duration was the subject of dispute throughout the years, and its administration ranged from 3 months in Denmark (Lebech et al., 1999) to 2 years in some French (Villena et al., 1998) and Swiss (Signorell et al., 1996) centers. The Danish neonatal screening program came up with the results of a 3-year follow-up of 47 infected children treated for 3 months with PYR-SDZ: of the 12 children with clinical CT at birth, only one had new eye lesions at age 1 and no new lesions were detected at the age 3 (Schmidt et al., 2006). These results were even better than the results of a US-based 10-year follow-up study of 327 neonates (24% had at least one eye lesion), who were treated with PYR-SDZ for one year: 29% had at least one new lesion after 10 years (Mets et al., 1996), but this discrepancy could be due to different epidemiological characteristics (lower proportion of avirulent type II strains in Northern America). As the risk of developing eye lesions was shown to be maximal when cerebral calcifications are present at birth (Kieffer et al., 2008), it has been proposed that treatment could be shortened to a 3-month course in asymptomatic neonates (Freeman et al., 2008). This alleviation of regimen would not be recommended outside Europe, as eye prognosis is tightly linked to parasite genotype (Pfaff et al., 2014).

#### 2.1.3 Adverse effects and resistance issues

PYR and SDZ are inhibitors of DNA synthesis in *T. gondii* tachyzoites but may also inhibit DNA synthesis in tissues with a high metabolic activity such as the bone marrow and epithelium. This can be bypassed by the addition of folinic acid (FA), and these adverse events are reversed upon cessation of treatment. In a recent randomized clinical trial on MFTP reduction by Mandelbrot et al. (Mandelbrot et al., 2018), no hematological toxicity was observed in 72 women treated with PYR-SDZ-FA, nor in the previous studies by Hotop et al. (119 pregnant women) and Prusa et al. (Hotop et al., 2012; Prusa et al., 2015). A systematic review showed that, of a total of 13 reported adverse events compiled in all studies focusing on PYR-based treatment of CT, 8 required discontinuation or change of treatment (Ben-Harari et al., 2017). Bone marrow suppression in treated infants and pregnant women was reported in 10 (76.9%) (McLeod et al., 2006) and one studies, respectively. In treated children, neutropenia, as well as anemia, were reported in 53.8% (7/13) studies, thrombocytopenia in 23.1% (3/13) and eosinophilia in 7.7% (1/13) (Ben-Harari et al., 2017).

The potential severity of adverse events of the PYR-SDZ combination led to considering alternative treatment options for CT, such as PYR-clindamycin, PYR-azithromycin, atovaquone, cotrimoxazole (TMP-SMX). However, clinical evaluation studies, preferably randomized, are urgently needed; a single study to date has shown a significant effect of TMP-SMX on the reduction of MFTP when combined with SPI, which was equivalent to that of PYR-SDZ (Valentini et al., 2009).

Although lack of compliance due to adverse events could explain treatment failures, several authors have reported possible resistance issues during the treatment of toxoplasmic encephalitis, chorioretinitis, and congenital toxoplasmosis (Dannemann et al., 1992; Katlama et al., 1996; Torres et al., 1997; Baatz et al., 2006; Petersen, 2007). Meneceur et al. studied 17 *Toxoplasma* strains of various genotypes and found several mutations on the DHFR gene, which were not linked to lower susceptibility to pyrimethamine (Meneceur et al., 2008). A higher IC50s variability was observed for sulfadiazine, ranging between 3 and 18.9 mg/L for 13 strains and >50 mg/L for three strains. More recently, Doliwa et al. used a proteomic approach by difference-gel electrophoresis combined with mass spectrometry to identify proteins that would be differentially expressed in sulfadiazine-resistant strains, compared to sensitive strains (Doliwa et al., 2013). They found that 44% of proteins were over-expressed in resistant strains and 56% were over-expressed in sensitive strains. The virulence-associated rhoptry protein, ROP2A, was found in greater abundance in both naturally resistant Type II strains TgH 32,006 and TgH 32,045 compared to the sensitive strain ME-49. Clearly, more studies are needed to determine whether resistance to sulfadiazine is linked to virulence of the parasite strain or to specific mutations.

### 2.2 Immunocompromised patients

Toxoplasmosis is a severe opportunistic infection in immunocompromised patients, resulting most often from the reactivation of dormant cysts in patients with chronic infection. Toxoplasmic encephalitis (TE) is the most common clinical manifestation which is observed with a high prevalence in HIV-infected patients, but the spectrum of disease has changed with the increasing number of non-HIV immunocompromised patients (Robert-Gangneux et al., 2015). Indeed, in non-HIV patients, the outcome is poorer, and toxoplasmosis is more often disseminated than confined to the CNS. It is particularly life-threatening in bone marrow or hematopoietic stem cell transplant (HSCT) patients, as mortality ranges from 38% to 67% despite treatment (Gajurel et al., 2015). Therefore, curative treatments for toxoplasmosis must be highly and quickly effective, and cross the blood brain barrier. All current treatment protocols have been established in HIV-infected patients, who represented the largest cohorts in the 1980s. Actually, the drugs used are the same as for congenital toxoplasmosis, underlining the small arsenal currently available, and the urgent need for new drugs.

Several clinical trials comparing curative treatments have been conducted in HIV+ patients presenting with TE (Table 2). In these studies, PYR/SDZ was evaluated at different regimens (PYR ranging from 50 mg to 100 mg/day) and/or compared to PYR/clindamycine (CLD) (Dannemann et al., 1992) or trimetoprime (TMP)/sulfamethoxazole (SMX) (Torre et al., 1998). Altogether, no clear difference was observed between treatment arms at the end of treatment (Table 2). PYR/SDZ seemed to be the most efficient treatment but the study power cannot show a superior treatment (Katlama et al., 1996), with fewer relapses during maintenance therapy than PYR/CLD, but it induced more side effects than PYR/CLD or TMP/SMX (Katlama et al., 1996; Torre et al., 1998).

**Table 2:** Comparative trials of curative treatments for toxoplasmosis in immunocompromised patients.

Clinical setting	Patients (No, type)	Induction regimen	Maintenance regimen	Duration (weeks)	Country	Result	Reference
TE	299 adults HIV+	P (50 mg/d) + Sz (4 g/d) vs P (50 mg/d) + C (2.4 mg/d)	P (25 mg/d) + Sz (2 g/d) vs P (25 mg/d) + C (1.2 g/d)	I: 6 M: Lifelong	Europe	Endpoint at 6 weeks: No superior treatment More relapses in P/C arm	(Katlama et al., 1996)
TE	77 adults HIV +	P (50 mg/d) + Sz (60 mg/kg/d) vs T (10 mg/kg/d) + Sx (50 mg/kg/d)	P (25 mg/d) + Sz (30 mg/kg/d) vs T (5 mg/kg/d) + Sx (25 mg/kg/d)	I: 4 M: 12	Europe (Italy)	No superior treatment More side effects in P/Sz arm	(Torre et al., 1998)
TE	59 Adults HIV+	P (200 mg D1; 75 mg/d) + Sz (100 mg/kg/d) vs P (200 mg D1; 75 mg/d) + C (4.8 g/d)		6	USA	Endpoint at 6 weeks No superior treatment	(Dannemann et al., 1992)
TE	30 adults HIV +	P (50 mg/d) + Sz (4 g/d) vs P (100 mg/d) + Sz (4 g/d) vs T (10 mg/kg/d) + Sx (50 mg/kg/d)		6	Thailand	Endpoint at 6 weeks More deaths in T/Sx group, but only 10 patients per group	(Kongsaengdao et al., 2008)
TE	41 adults HIV+	P (200 mg D1; 50 mg/d if <60 kg or 75 mg/d if >60 kg) + Sz (4 g/d) vs T (20 mg/kg/d) + Sx (100 mg/kg/d) + C (1.8 g/d)	P (25 mg/d) + Sz (2 g/d) vs T (10 mg/kg/d) + Sx (50 mg/kg/d) + C (0.9 g/d)	I: 4–6 M: until CD_4_ count >200/mm^3^	India	More complete responses and less relapses in T/S + C group	(Goswami et al., 2015)
TE	39 adults HIV+	A (3 g/d) + P (200 mg D1; 50 mg/d if <60 kg or 75 mg/d if >60 kg) vs A (3 g/d) + Sz (4 g/d if<60 kg or 6 g/d if >60 kg)		I: 6 M: 42	Europe and USA	No superior treatment 75% vs 82% of responses at 6 weeks	(Chirgwin et al., 2002)
OT	149, No AIDS but HIV status unknown, 2 IS	P (100 mg D1; 50 mg/d) + Sz (4 g/d) + CS (60 mg/d) vs C (1.8 g/d) + Sz (4 g/d) + CS (60 mg/d) vs T (320 mg/d) + Sx (1600 mg/d) + CS (60 mg/d)		4	Europe (The Netherlands)	P/Sz + CS: reduction of retinal lesions compared with other combinations	(Rothova et al., 1993)

TE: Toxoplasmic encephalitis; OT: Ocular toxoplasmosis; HIV: Human immunodeficiency virus; IS: Immunosuppressed patient; P: Pyrimethamine; Sz: Sulfadiazine; C: Clindamycin; T: Trimetoprime; Sx: Sulfamethoxazole; CS: Corticosteroids; A: Atovaquone; vs: versus; I; induction; M: maintenance.

American (Kaplan et al., 2009) and European guidelines (http://www.eacsociety.org/files/2018guidelines-9.1-english.pdf) on treatment of opportunistic infections in patients living with HIV are based upon those studies and grade recommendations according to evidence-based medicine criteria proposed by the Infectious Diseases Society of America (IDSA) (Table 3), with at least a 6 week-induction treatment (until clinical and radiological improvement). The preferred regimen is PYR (200 mg loading dose on day 1, then 75 mg/day if patient weighs ≥60 kg or 50 mg/day if patient weighs <60 kg), associated with (SDZ) (3000 mg twice a day if patient weighs ≥60 kg or 2000 mg twice a day if patient weighs ≥60 kg) and folinic acid (10 to 15 mg/day) (grade **AI** recommendation). Alternatives therapies are PYR/CLD (600 to 900 mg four times a day) + folinic acid (grade **AI**) (Dannemann et al., 1992; Katlama et al., 1996) or TMP (5 mg/kg twice a day)/SMX (25 mg/kg twice a day) (grade **BI**) (Torre et al., 1998). If patient cannot tolerate those molecules, atovaquone (1500 mg twice a day) can be used in association with pyrimethamine (and folinic acid) or with sulfadiazine (Chirgwin Chirgwin et al., 2002), grade **BII**) and azithromycin (900 to 1200 mg/j) can be associated with pyrimethamine (and folinic acid) (Saba et al., 1993; Jacobson et al., 2001), grade **BII**.

**Table 3:** Rating scheme for treatment recommendations according to IDSA.

Category	Definition
A	Both strong evidence for efficacy and substantial clinical benefit support recommendation for use. Should always be offered.
B	Moderate evidence for efficacy – or strong evidence for efficacy but only limited clinical benefit – supports recommendation for use. Should generally be offered.
C	Evidence for efficacy is insufficient to support a recommendation for or against use. Or evidence for efficacy might not outweigh adverse consequences (e.g. drug toxicity, drug interactions) or cost of the treatment or alternative approaches. Optional.
D	Moderate evidence for lack of efficacy or for adverse outcome supports a recommendation against use. Should generally not be offered.
E	Good evidence for lack of efficacy or for adverse outcome supports a recommendation against use. Should never be offered

Sub-category	Quality of the evidence supporting the recommendation

I	Evidence from at least one properly-designed randomized, controlled trial.
II	Evidence from at least one well-designed clinical trial without randomization, from cohort or case-controlled analytic studies (preferably from more than one center), or from multiple time-series studies, or dramatic results from uncontrolled experiments.
III	Evidence from opinions of respected authorities based on clinical experience, descriptive studies, or reports of expert committees.

Along with side effects, mainly myelotoxicity, the therapeutic choice can be guided by availability or price of molecules. Indeed, pyrimethamine is unavailable and/or unaffordable in many countries, thus explaining the wide use of TMP/SMX as first line treatment in these countries (Gallant, 2015; Goswami et al., 2015). PYR/SDZ and TMP/SMX present good biodisponibility (70% and 90% respectively) and all these molecules have plasmatic half-life around 10 h, making possible to obtain peak concentration after only 2 to 6 h apart from pyrimethamine that has a long half-life (around 4 days) underlying the need for a loading dose. In addition to that the biodisponibility of pyrimethamine decrease in malnourish patient (de Kock et al., 2018) making TMP/SMX a first choice in developing countries. Recent reviews and meta-analysis (Rajapakse et al., 2013; Wei et al., 2015; Hernandez et al., 2017) were not able to show a superior antiparasitic association among PYR/SDZ, PYR/CLD or TMP/SMX, hence reinforces the possibility to choose TMP/SMX. Furthermore, TMP/SMX is the only intravenous regimen when oral route is not practicable. Taken together, while guidelines recommend PYR/SDZ, TMP/SMX can be a valuable and convenient alternative in low income countries. However, TMP/SMX is also the only available efficient combination for prophylaxis in patients at risk for *Toxoplasma* reactivation (CD4+ T cells <200/μL). Thus the wide use of the same drug for both prophylaxis and curative treatments is awkward, as it could favor the emergence of resistance in the long term.

In TE, adjunction of corticosteroid can be necessary to improve brain edema (Luft et al., 1993), but it can be sometimes detrimental by increasing immunosupression (Arens et al., 2007) and differential diagnosis of lymphoma may be difficult after corticotherapy.

After induction therapy, maintenance therapy/secondary prophylaxis is introduced. For HIV patient, this treatment is maintained until CD4 count stays above 200 cells/mm^3^ and HIV viral load is undetectable for up to 6 month (Kaplan et al., 2009; European AIDS Clinical Society (EACS), 2018). All curative regimens with lower doses can be used, PYR/SDZ + FA; PYR/CLD + FA; Atovaquone alone or associated with PYR + FA; or TMP/SMX. But PYR/CLD have no effect on *Pneumocystis*, thus an additional prophylaxis is necessary to prevent *Pneumocystis jirovecii* pneumonia. As for induction treatment, price and side effects can orient therapeutic choice.

In HSCT patients, *Toxoplasma* reactivation is often disseminated, rather than cerebral or ocular (Robert-Gangneux et al., 2018), and occurs mainly in the first six months after transplantation (Gajurel et al., 2015). There are no therapeutic comparative trials in the literature, and in the absence of specific guidelines, those for HIV patients are followed for these patients. PYR/SDZ is the first choice treatment in 80% of cases, followed by TMP/SMX and PYR/CLD (Mele et al., 2002; Martino et al., 2005). Because of the severity of the disease, it has been suggested that a third antiparasitic drug could be added (PYR/SDZ + CLD or + spiramycin; TMP/SMX + CLD) (Mele et al., 2002). After solid organ transplantation, *Toxoplasma* infection occurs mostly through contamination by the organ of a seropositive donor containing dormant cysts, in a naïve recipient (Dhakal et al., 2018); heart transplant recipient are the most at risk in this setting (Gallino et al., 1996). However, it can be also a primary infection by oral route, irrespective of the kind of transplantation (Campbell et al., 2006; Fernàndez-Sabé et al., 2012; Robert-Gangneux et al., 2018). Like for HSCT patients, curative treatment usually follows HIV guidelines, with PYR/SDZ or TMP/SMX as first line therapy. Clindamycin, azithromycin and atovaquone are alternative treatments when PYR/SDZ or TMP/SMX cannot be used, despite the lack of randomized studies to support their use. In bone marrow or solid organ transplantation, lower or discontinued immunosuppression plays an important role in the outcome, as immune restoration is part of treatment success (Campbell et al., 2006; Chung et al., 2008).

Systemic treatment (Butler et al., 2013; Ozgonul and Besirli, 2017) combining antiparasitic drugs and corticosteroids (Holland and Lewis, 2002) is recommended for ocular toxoplasmosis in immunocompromised patients. However, comparative studies in this patient population are scarce. One was conducted in patients with unknown HIV status (Rothova et al., 1993); another one excluded immunosuppressed patients (Bosch-Driessen et al., 2002). Despite this lack of clinical trials, the classical PYR/SDZ regimen associated with corticosteroids is considered as the treatment of choice (Rothova et al., 1993; Holland and Lewis, 2002; Butler et al., 2013). Other antibiotics can be used, such as azithromycin (Bosch-Driessen et al., 2002), or clindamycin, spiramycin (Rothova et al., 1993) but no treatment showed superiority.

## 3 Future therapeutic perspectives

### 3.1 New candidate molecules

Searching for anti-*T. gondii* compounds is a difficult task, since it should be active on both tachyzoites and bradyzoite stages. Currently, no ideal drug is available, but efforts into developing promising drug candidates are continuously ongoing (Escotte-Binet et al., 2018). A number of preclinical studies (in vitro and in vivo) have been conducted and some compounds demonstrated low IC50, a high selectivity index in vitro, prolonged survival of infected animals in murine models of acute toxoplasmosis, and reduced parasite loads in brain and muscle tissues in models of chronic toxoplasmosis (McFarland et al., 2016).

Two different approaches in the drug discovery process will be discussed in this paper, i) searching for compounds with effects on specific parasitic targets, and ii) repurposing of drugs or promising compounds active against other pathogens. These two approaches often overlap. Known or suggested mechanisms of action and targets of reviewed drugs and compounds are presented in Table 4.

**Table 4:** Overview of novel or re-purposed drugs with anti-*Toxoplasma* activity.

Drug/compound	Mechanism of action	Target	Activity			Reference
			In vitro	In vivo*-*tachyzoites	In vivo-bradyzoites	
Dihydrotriazine JPC-2067-B	Folate pathway	Dihydrofolate reductase	+	+	ND	(Mui et al., 2008)
JPC-2056			ND	+	ND	(Mui et al., 2008)
MMV675968^a^			+	ND	ND	(Spalenka et al., 2018)
Atovaquone	Electron transport pathway	Cytochrome bc1	+	+	+	(Romand et al., 1993; Ferguson et al., 1994) (Djurković-Djaković et al., 1999, Djurković-Djaković et al., 2002)^h^
Para-hydroxynaphthoquinones			+	+	+	(Ferreira et al., 2002, Ferreira et al., 2006)
Amino-terpenylnaphthoquinones (QUI-11, QUI-6, and QUI-5)			+	ND	+^g^	(Ferreira et al., 2012)
MMV689480 (buparvaquone)^a^			+	+^e^	ND	(Müller et al., 2017)^e^(Spalenka et al., 2018)
Endochin-like quinolones (ELQ-271, ELQ-316)			+	+	+	(Doggett et al., 2012)
Triclosan	Fatty acid synthesis II pathway	Enoyl-acetyl carrier protein reductase	ND	+	+	(El-Zawawy et al., 2015a, El-Zawawy et al., 2015b)
Thiolactomycin		β-ketoacyl-acyl carrier protein synthase	+	ND	ND	(Martins-Duarte et al., 2009)
Newly synthesized bisphosphonates	Isoprenoid pathway	Farnesyl diphosphate/geranyl geranyl-diphosphate synthase	+	+	ND	(Shubar et al., 2008)
Artemisone and artemiside	Ca-dependent pathway	Ca-ATPases	+	+	+	(Dunay et al., 2009)
Bumped kinase inhibitor 1294		Calcium-dependent protein kinase 1	+	+	ND	(Doggett et al., 2014) (Müller et al., 2017)^e^
Compound 32			+	+	+	(Vidadala et al., 2016)
Compound 24			+	+	+	(Rutaganira et al., 2017)
FR235222	Gene expression control	Histone deacetylase enzymes	+	ND	+^g^	(Maubon et al., 2010)
W363 and W399			+	ND	ND	(Maubon et al., 2010)
Rolipram	cAMP signaling pathways	Phosphodiestrase 4	ND	ND	+	(Afifi and Al-Rabia, 2015)
Guanabenz	Translational control	TgIF2α phosphorilation	+	+	+	(Benmerzouga et al., 2015)
MMV007791 (piperazine acetamide)^b^	Active against *Plasmodium*	Uncertain	+	ND	ND	(Boyom et al., 2014)
Newly synthesized quinoline compounds: 8-hydroxyquinoline and 4-aminoquinoline (B23)	Apicoplast level	Apicoplast metabolic functions	+	ND	ND	(Kadri et al., 2014)
Tanshinone IIA^c^	Anticancer	Uncertain	+	ND	ND	(Murata et al., 2017)
Hydroxyzine^c^	Histamine receptor antagonist					
Compounds with antiinflammatory and anticancer properties^d^	Antiinflammatory and anticancer	Uncertain	+	ND	ND	(Adeyemi et al., 2018)
Miltefosine	Anticancer	Enzymes involved in phospholipid metabolism	**+**	+^f^	+	(Ni Nyoman and Lüder, 2013; Eissa et al., 2015)
Tetraoxane (compound 21)		Uncertain	ND	+	ND	(Opsenica et al., 2015)

ND: not done.

a Compounds from Pathogen box provided by Medicines for Malaria Venture (MMV).

b Compound from Malaria box provided by Medicines for Malaria Venture (MMV).

c Compounds from chemical compound library provided by the Drug Discovery Initiative (University of Tokyo, Japan).

d Natural product library and FDA-approved drugs.

e Vertical transmission model.

f Poor effects.

g Evaluated in ex vivo model system.

h In combination with clindamycin.

Searching for pathways and processes that are essential for parasite survival focuses on essential metabolic pathways (Table 4). Attractive drug targets would be metabolic pathways that are in charge of synthesis of components that are not supplied by the host cell (fatty acids, sterols and polyisoprenoids…). Ideally, these pathways should be absent or different from the corresponding mammalian pathways. In particular, drugs that affect processes important for gliding motility, host cell invasion and egress, or that interfere with the parasite differentiation process have been reported as promising ones (Alday and Doggett, 2017). Pathways affected by currently used drugs (folate pathway and electron transport pathway) are still considered by researchers in their efforts to overcome drug resistance and find more effective and safe drugs. PYR analogs that would target specifically the parasite DHFR, would avoid the adverse effects caused by inhibition of the host folate mechanism. An extremely promising novel compound among the DHFR inhibitors is dihydrotriazine JPC-2067-B87, previously investigated as an antimalarial candidate, which showed to be highly effective against *T. gondii* in vitro with a low IC50 value. Its prodrug, JPC-2056, was tested in a murine model of acute toxoplasmosis and was effective when administered orally (Mui et al., 2008). However, its effect on cysts is not yet known and needs to be investigated.

The cytochrome *bc*1 complex is also a frequent drug target against Apicomplexa, including *T. gondii* (Alday and Doggett, 2017) (Table 4). Cytochrome bc1 complex inhibitors bind to the Qo or Qi site of the complex and disrupt cell respiration by acting on the electron transport pathway (Docampo et al., 1978). The only clinically used cytochrome *bc*1 inhibitor is atovaquone, which, like other naphthoquinones, binds to the Qo site. Its activity on tissue cyst burden was demonstrated both when used alone (Ferguson et al., 1994), and combined with clindamycin (Djurković-Djaković et al., 2002). Unfortunately, mutations of the binding site of this drug on cytochrome *b* can occur, leading to development of resistance and limiting its wide use (McFadden et al., 2000). Ferreira et al. (2002) showed significant in vitro inhibitory effect of seven naphthoquinones, three of which (para-hydroxynaphthoquinones (PHNQ) prolonged survival of mice infected with a virulent *T. gondii* strain (Ferreira et al., 2002). One of them, the PHNQ6 compound, was further evaluated in a chronic infection model. It was administered alone and in combination with sulfadiazine 30 days after infection with an avirulent strain, and resulted in reduction of brain cyst burdens in both cases compared to control mice (Ferreira et al., 2006). The same group also investigated the anti-*T. gondii* properties of new amino-terpenylnaphthoquinones (QUI-11, QUI-6, and QUI-5). In vitro incubation of *T. gondii* cysts with QUI-6 and QUI-11 resulted in inhibition of bradyzoite infectivity, since none of the surviving animals inoculated with treated cysts had detectable cysts in their brains (Ferreira et al., 2012). Unlike naphthoquinones which bind to the Qo site of the bc1 complex, another class of compounds worth mentioning is a group of 4-(1H)-quinolone derivatives with endochin-like quinolones (ELQs) hypothesized to bind to the other site of cytochrome *bc*1 complex, the Qi site. From a library of 4(1H)-quinolone-3-diarylethers, ELQ-271 and ELQ-316 were selected for further investigation and showed low IC50s. These compounds were significantly more effective than atovaquone in a murine model of acute toxoplasmosis, reducing the number of brain cysts by 88% in mice treated five weeks after infection with the ME49 strain (Doggett et al., 2012). Since atovaquone and ELQs act on different sites (Qo and Qi) of the same complex, it has been suggested that a combination of these two compounds would act synergistically on the parasite bc1 complex and prevent the development of resistance (Alday and Doggett, 2017).

Several important *T. gondii* metabolic pathways take place in the apicoplast, making this organelle of interest as a potential drug target (Montazeri et al., 2017). One potential new drug target is the enoyl-acetyl carrier protein reductase (ENR) of the type II fatty acid synthesis (FAS II) pathway (Ramakrishnan et al., 2015). Despite the ability of *T. gondii* to scavenge some fatty acids from the host cell, those produced in the apicoplast are required for organelle biogenesis (Mazumdar et al., 2006). An antibacterial drug which inhibits ENR, triclosan (5-chloro-2-[2,4-dichlorophenoxy] phenol), was investigated in murine models of acute and chronic toxoplasmosis (El-Zawawy et al., 2015a, El-Zawawy et al., 2015b), and showed a reduction of burden and of viability of both tachyzoites and cysts. Another drug that interferes with the same pathway is thiolactomycin. It specifically inhibits β-ketoacyl-acyl carrier protein synthase, another enzyme essential for elongation of fatty acids (Jackowski et al., 1989). Among eight thiolactomycin analogs, all but one showed better activity against *T. gondii* tachyzoites in vitro compared to the parent compound. The two analogs with longer alkyl chains at the C5 position had the lowest IC50s and caused changes in parasite morphology, suggesting the importance of the length of the compound side group for its interactions with the drug target (Martins-Duarte et al., 2009). However, these derivatives have not yet been tested for in vivo activity.

The apicoplast isoprenoid pathway is also important for parasite survival, with the bifunctional enzyme farnesyl diphosphate/geranyl geranyl-diphosphate synthase being crucial for synthesis of sterols and polyisoprenoid compounds. A series of newly synthesized bisphosphonates, which interfere with the isoprenoid pathway through inhibition of this enzyme, showed low toxicity and excellent activity against *T. gondii* in vitro and in vivo (Shubar et al., 2008).

The calcium-dependent pathway, regulated by calcium-dependent protein kinase 1 (TgCDPK1) which differs from human kinases, controls processes essential for *T. gondii* gliding motility, host-cell invasion and egress (Lourido et al., 2010). Among highly promising TgCDPK1 inhibitors, pyrazolo-pyrimidine (PP)-based compounds showed good efficacy in murine models of toxoplasmosis (Alday and Doggett, 2017). However, further evaluation of a promising compound, bumped kinase inhibitor BKI 1294, was interrupted (Doggett et al., 2014) because of the suspected risk of cardiotoxicity through the inhibition of the human Ether-à-go-go-Related Gene (hERG) ion channel (Ojo et al., 2014). Nevertheless, Vidadala et al. optimized a new compound (compound 32) with modifications in the main PP scaffold that retained favorable BKI 1294 properties, but lacked the hERG inhibitory activity (Vidadala et al., 2016). This compound significantly reduced the parasite burden in a murine model of acute *T. gondii* infection. Moreover, it was able to penetrate the CNS where it significantly reduced the brain cyst burden (by 88.7%) when administered five weeks after infection. Rutaganeira et al., conducted further investigation of PP-based CDPK1 inhibitors in murine models; treatment with compound 24 during the first days of infection allowed reduction of the brain cyst burden in surviving mice at day 30, but more importantly, administration of this compound after removal of sulfadiazine in immunocompromised (IFNγ receptor-deficient) mice resulted either in a complete cure or a delay in the reactivation of chronic toxoplasmosis (Rutaganira et al., 2017).

Parasite interconversion between tachyzoite and bradyzoite stages is a fundamental event in the pathogenesis of toxoplasmosis. A blockade of the differentiation into bradyzoites could help cure an acute infection, whereas a blockade of the differentiation into tachyzoites process would be of great importance for chronically infected immunocompromised patients, who are at risk of reactivation. Histone acetylase (HAT) and histone deacetylase (HDAC) enzymes control histone acetylation levels, and have an important role in the control of gene expression during parasite interconversion. It is hypothesized that inhibitors of *T. gondii* HDAC3 (TgHDAC3) disrupt the usual level of histone acetylation across the genome (Bougdour et al., 2009; Maubon et al., 2010). One TgHDAC3 inhibitor, the cyclopeptide FR235222, inhibited *T. gondii* intracellular growth, induced conversion of tachyzoites to bradyzoites in vitro (Bougdour et al., 2009), and even reached bradyzoites within ex vivo cysts, making them incapable of converting again into tachyzoites. Also, when FR235222-treated cysts were inoculated in mice, they were incapable of causing infection. Two derivatives of FR235222, W363 and W399, were further evaluated in vitro and showed IC50s equivalent to that of the parent compound, while being less cytotoxic (Maubon et al., 2010). Effectiveness of FR235222 and derivatives against chronically infected mice remains to be demonstrated in vivo.

Phosphodiestrase-4 (PDE4) inhibitors interfere with tachyzoite to bradyzoite interconversion by affecting the cAMP signaling pathways, and probably by suppression of pro-inflammatory Th1 cytokines. (Afifi and Al-Rabia, 2015) showed that one PDE4 inhibitor, rolipram, allowed for a 74% reduction of the brain cyst load in chronically infected mice. Unfortunately, severe nausea and vomiting caused by this drug were reported (O'Donnell and Zhang, 2004).

Translational control via phosphorylation of the *T. gondii* eukaryotic initiation factor 2 (TgeIF2) is essential in both the tachyzoite and bradyzoite stages. Recently, it has been shown that guanabenz, an FDA-approved antihypertensive drug, interferes with translational control through inhibition of eIF2 dephosphorylation, without involving host's eIF2. Guanabenz showed activity on both stages of the parasite, i.e. it was able to protect mice against acute toxoplasmosis, but also to cross the blood-brain barrier and reduce the number of brain cysts (up to 69%) in chronically infected mice (Benmerzouga et al., 2015).

Aside from well-known drug targets, an interesting novel approach using morpholinos for identifying and characterizing new *T. gondii* targets of therapeutic interest has recently been described (Lai et al., 2012; McPhillie et al., 2016). Lykins et al. used modified forms of morpholinos with covalently attached chemical groups to facilitate entry into cells (vivoPMO), in order to decrease the expression of specific *T. gondii* enzymes (Lykins et al., 2018). This approach may identify enzymes potentially important in parasitic replication and thus attractive as new drug targets.

The other frequent approach in the drug discovery process is screening of a wide range of available chemical compounds. Studies focusing on drug repurposing are conducted, as it was noticed that compounds active against one apicomplexan parasite are often active against other apicomplexans, possibly in relation with conserved biochemical pathways, and consequently common targets.

Artemisinin, a highly potent antimalarial, as well as its numerous derivatives produced over years, showed potential in the treatment of murine toxoplasmosis (Alday and Doggett, 2017). Particularly interesting are artemisone and artemiside, which prolonged survival and reduced the brain cyst burden in mice infected with tachyzoites of a type II *Toxoplasma* strain. They also prolonged survival in a murine model of reactivated toxoplasmosis, but all the animals died following discontinuation of drug administration, indicating that artemisinin derivatives acts on tachyzoites, but not on bradyzoites (Dunay et al., 2009).

The availability of chemical compound libraries allows for high throughput screenings against *T. gondii*. For instance, Boyom et al. conducted in vitro drug screening of the open-access Medicines for Malaria Venture (MMV) Malaria Box containing 400 blood-stage-active anti-*Plasmodium* compounds (Boyom et al., 2014). This screening led to the identification of seven potent anti-*T. gondii* compounds, the most potent and selective of which was MMV007791, a piperazineacetamide. These compounds had novel chemical scaffolds that differed from the pyrimidine present in available drugs for toxoplasmosis. A similar open-access library called Pathogen box consisting of 400 compounds with activity against diverse pathogens, was subjected to anti-*T. gondii* screening by Spalenka et al. (Spalenka et al., 2018). Fifteen compounds turned out to be effective, with eight having both selective and favorable in vitro effects on the growth of tachyzoites. Out of the three most active compounds, MMV675968 was previously described as having an anti-*Cryptosporidium* effect targeting DHFR, and it is probable that it also targets DHFR in *Toxoplasma*. Another one was buparvaquone, a well-known hydroxynaphthoquinone, previously shown to inhibit *Neospora caninum* proliferation by inhibition of enzymes involved in the mitochondrial electron transport (Spalenka et al., 2018).

While screening of the Malaria and the Pathogen boxes focused on early preclinical leads, a recent study by Radke et al. (2018) evaluated currently approved antimalarial drugs and drugs in late preclinical development (Radke et al., 2018). Surprisingly, the majority of these drugs showed limited activity against *T. gondii* in vitro. Particularly, 4-amino and 8-aminoquinolines derivatives had modest or even very little potency in inhibiting *T. gondii*, while, Kadri et al. (2014) had previously reported promising results with newly synthesized quinoline compounds and one 4-aminoquinoline (Kadri et al., 2014).

Screening of a chemical compound library provided by the Drug Discovery Initiative (University of Tokyo, Japan) revealed two effective inhibitors in vitro, without causing host cell toxicity, i.e. tanshinone IIA (a compound that may inhibit cancer cell growth), and the antihistamine drug hydroxyzine. More interestingly, these compounds showed inhibitory effects on the growth of intermediately differentiated bradyzoites, a property lacking in current drugs (Murata et al., 2017).

Finally, Adeyemi et al. screened a large natural products library, as well as a library of FDA-approved drugs with various treatment indications (Adeyemi et al., 2018). The majority of the compounds that inhibited parasite growth had anti-inflammatory and anti-cancer properties. This was not surprising, as it was already demonstrated that some drugs used in cancer and inflammatory therapy, showed potent activity on *T. gondii* growth in vitro (Dittmar et al., 2016; Murata et al., 2017), but also on *Leishmania* growth. Of them, miltefosine showed promising results in treating numerous protozoal infections, and is now widely used in the treatment of visceral leishmaniasis. Its ability to successfully treat encephalitis caused by free living amoebas emphasizes its potential for crossing the blood-brain barrier (Schuster et al., 2006; Webster et al., 2012). It did not show potency in controlling acute toxoplasmosis, but conversely, administration of this drug 60 days after murine infection with ME49 strain, led to a reduction of the brain cyst number and size (Eissa et al., 2015). Another anti-cancer molecule (tetraoxane) was tested in a murine model of acute toxoplasmosis and significantly prolonged survival of infected mice compared to control mice (Opsenica et al., 2015).

Overall, these studies are encouraging and show that the arsenal of drug candidates is expanding, with some of them being very promising. However, further studies are needed in vivo to confirm results obtained in vitro.

### 3.2 Immunotherapeutic strategies against *Toxoplasma* infection: sound or unrealistic?

Toxoplasma acute infection remains largely asymptomatic in immunocompetent hosts, thanks to an appropriate immune control resulting in parasite encystation. However, partial subversion of the host response by the parasite allows lifelong persistence of cysts. This host-parasite cohabitation relies on a tightly regulated balance, to control parasite replication. The occurrence of any immune defect, particularly affecting the T-cell response, mostly secondary to HIV infection or immunosuppressive therapies, results in parasite reactivation, leading to severe encephalitis or disseminated infection (Hegab and Al-Mutawa, 2003). Currently, none of the therapies described above are able to eradicate the parasite from infected hosts, leaving them vulnerable to further relapses. In this context, for many years, immunotherapy has emerged as a promising and beneficial approach for the management of toxoplasmosis, allowing immunocompromised hosts to recover an adequate immune response (Fung and Kirschenbaum, 1996). Continuous advances in the understanding of toxoplasmosis immunopathogenesis have given new insights on how to counteract immune impairments. Apart from vaccine development which raises many active researches (Lim and Othman, 2014; Rezaei et al., 2018) and are addressed in a review in this special issue (Innes et al., 2019), alternative immunomodulation strategies, including adoptive cell transfer and passive immunization will be successively discussed.

The immune response against *T. gondii*, involves complex mechanisms of both innate and adaptive immunity. While innate effectors play an important role during acute infection (Yarovinsky, 2014), long-term protection is mediated by the adaptive immune response. In particular, cellular effectors, mainly IFNγ-producing T lymphocytes, are needed to control multiplication and spread of *T. gondii*, and maintain latency (Khan et al., 1999). Especially, CD8+ T cells and NK cells act through the lysis of infected cells, and consequently render the parasite accessible to other immunological mechanisms, including immunoglobulins, complement, macrophages and dendritic cells (Bhadra et al., 2011a; Yarovinsky, 2014). Th1 pro-inflammatory response involving cytokines such as IL-12, IFNγ, TNFα, as well as IL-7 and IL-15 is the mainstay of an adequate anti-parasitic response, initially during acute infection to reduce parasite burden, and then during chronic infection to exert immune pressure, sufficient enough to maintain parasite encystment (Gazzinelli et al., 1993; Khan et al., 1994; Khan and Kasper, 1996; Yap et al., 2000; Bhadra et al., 2010). Conversely, immunosuppressive cytokines such as IL-10, contribute to the parasite intracerebral persistence, and the impairment of local immune response leads to the reactivation of latent *T. gondii* cysts in the brain (Khan et al., 1995).

Firstly, to reinforce innate immunity, the administration of interleukin-18 was shown to enhance IL-12-mediated resistance to *Toxoplasma* in profoundly immunosuppressed SCID mice, leading to reduced parasite burdens and delayed time to death (Cai et al., 2000). Although endogenous IL-18 appeared to have limited involvement in innate resistance, the protective role conferred by exogenous IL-18 supplementation was correlated with increased NK cell numbers and cytotoxic activity, together with elevated levels of INOS in splenic tissue.

Another strategy could aim at counteracting the biological activity of deleterious cytokines, as proposed by Deckert-Schlüter et al. (1997), who assessed the relevance of anti-IL-10 antibodies in a mice model of chronic encephalitis (Deckert-Schlüter et al., 1997). Interestingly, it led to the decrease of intracerebral parasitic load, related to an enhanced expression of IFNγ and TNFα, and a higher intracerebral recruitment of CD4+ and CD8+ T cells. However, IL-10-deficient mice quickly succumbed to necrotizing hepatitis, resulting from CD4+ T cell-mediated immunopathology with overproduction of IFNγ, IL-12 and TNFα (Gazzinelli et al., 1996). This study underlined the complexity of developing such interventions which can be dampened by adverse secondary effects.

Another major target for immunotherapeutic interventions is the CD8 T-road, as the loss of CD8+ T cell functionality (including optimal production of IFNγ) is a well-recognized feature responsible for parasite reactivation (Bhadra et al., 2011b; Bhadra et al., 2012). Precisely, this CD8 “exhaustion” is related to the overexpression of the inhibitory lymphocyte receptor PD-1 (Programmed Death-1) on T cell surface, leading to elevated apoptosis and progressive attrition of their functions. Logically, the blockade of PD-1/PDL-1 pathway by inhibitory antibodies has been explored and revealed an interesting rescue of dysfunctional CD8+ T cells (Bhadra et al., 2011b). Chronically infected mice significantly improved the control of parasite recrudescence (lower rate of *Toxoplasma*-infected leukocytes in brain and blood), leading to an enhanced survival, compared to untreated animals. However, such strategies should be cautiously considered, given that a prolonged blockade of PD-1 carries the risk of autoimmunity (Kasagi et al., 2011).

In the new era of cellular-based therapies, the adoptive transfer of immune CD8+ cells has been proposed, especially in the context of chronic *Toxoplasma* infection. While it showed to temporarily restrict the breakdown of cysts and confer a transient protection against the parasite reactivation (within four weeks post-treatment), it did not allow a long-term rescue of exhausted T cells, because donor cells failed to become long-lived (Bhadra et al., 2013). Thus this strategy could be only considered as prevention therapy of reactivation in high-risk immunocompromised patients.

Besides, regarding immunotherapy interventions, despite many advances in the field of vaccination, the efficiency of immunization therapies may be hampered in immunocompromised hosts, due to reduced cellular immunity. Thus, this issue raised interest for passive immunization assays, that could confer at least transitorily, an adequate protection against reactivation (Lim and Othman, 2014). Indeed, the use of specific recombinant anti-*Toxoplasma* antibodies represents a promising strategy, to block at early stage, parasite attachment and host cells invasion (Cha et al., 2001; Fu et al., 2011), or intracellular tachyzoite replication (Tan et al., 2010). Among others, antibodies against SAG1 antigen, the most abundant and immunogenic antigen at the parasite surface, aroused many expectations. Especially, Fab antibody fragments to SAG1 were shown to increase by 50% the survival in mice after lethal challenge, by direct blockade of cell invasion, rather than promoting Fc-dependent phagocytosis, cellular cytotoxicity or complement activation (Fu et al., 2011). Thus, by slowing parasite invasion, such therapies could be considered as prophylactic or adjuvant therapies combined to curative anti-parasitic treatments in immunocompromised hosts.

Taken together, several different approaches could be suitable for the control of *Toxoplasma* infection in immunocompromised patients, but have been mostly explored in mouse models, thus clinical trials in humans are still lacking to validate such strategies. Immunomodulation interventions for toxoplasmosis control could be fueled by researches in the field of leishmaniasis. Indeed, *Leishmania* parasites are also intracellular parasites, able to escape the host immune response and cause chronic visceral disease, all the more serious and fatal in immunocompromised hosts. The poor therapeutic response to conventional treatment could be compensated by the adjunction of alternative therapy, including supplementation or inhibition of specific interleukins (anti-IL-10), indirect immunostimulatory drugs, cellular or antibody transfers, as recently reviewed (Adriaensen et al., 2018). Recently the immunomodulatory properties of nanochitosan particles gave rise to renewed interest for the treatment of cutaneous leishmaniasis, alone or as adjuvant therapy (Bahrami et al., 2015; You et al., 2017). Interestingly, Teimouri et al. showed that nanochitosan particles alone were at least as efficient as sulfadiazine against *Toxoplasma* RH tachyzoites in vitro (Teimouri et al., 2018), and Etewa et al. reported reduced mortality in RH strain-infected mice treated with nanochitosan particles loaded with spiramycin (Etewa et al., 2018).

Thus, the boost of the host immune response could be a seducing therapeutic approach 1) to prevent reactivation of toxoplasmosis in immunocompromised patients if it has less adverse effects than the widely used drug cotrimoxazole, or 2) to help treating severe reactivation episodes in combination to conventional drugs, but active research in the field is still needed.
